# Spinal pain and nutrition in adolescents - an exploratory cross-sectional study

**DOI:** 10.1186/1471-2474-11-138

**Published:** 2010-06-30

**Authors:** Mark C Perry, Leon M Straker, Wendy H Oddy, Peter B O'Sullivan, Anne J Smith

**Affiliations:** 1 School of Physiotherapy and Curtin Health Innovation Research Institute, Curtin University of Technology, GPO Box U1987, Perth WA 6845, Perth, WA, Australia; 2 Centre for Sports and Exercise Medicine, Barts and the London School of Medicine and Dentistry, London, UK; 3 Telethon Institute for Child Health Research, Perth, WA, Australia; 4 Centre for Child Health Research, The University of Western Australia, Perth, WA, Australia

## Abstract

**Background:**

Spinal pain is an important health issue for adolescents resulting in functional limitations for many and increasing the risk of spinal pain in adulthood. Whilst human and animal studies suggest nutrition could influence spinal pain, this has not been investigated in adolescents. The objective of this exploratory cross sectional study was to evaluate associations between diet and adolescent spinal pain.

**Methods:**

This study surveyed the spinal pain (neck and back) and nutrition (specific nutrients, broad food groups, diet quality and dietary pattern) of 1424 male and female adolescents at 14 years of age, in Western Australia.

**Results:**

Back or neck pain were experienced by around half of the adolescents, with females more likely to experience spinal pain. Nutrition differed between sexes and deviated from optimal intakes. Vitamin B12, eggs, cereals and meat consumption were related to spinal pain in sex specific multivariate analyses including primary carer education level and adolescent waist girth and smoking.

**Conclusions:**

The findings of this study suggest that certain aspects of diet may have an association with spinal pain in adolescence.

## Background

Spinal pain is a serious problem for adolescents, with over half having experienced some form of back or neck pain by mid adolescence[1,2] and a third experiencing a reduction in function [3]. Adolescent spinal pain also appears to be a precursor to adult spinal pain [4-6], which has well-documented personal and societal costs [7]. Identification of contributing factors is therefore warranted.

Adolescent spinal pain has been shown to relate to physical factors such as obesity [8], psychosocial factors such as emotional problems [9] and lifestyle factors. Aspects of lifestyle such as very high or very low activity [10] or excessive computer use [11] have been shown to relate to adolescent spinal pain in cross-sectional studies. Moreover two longitudinal studies have shown links between adolescent spinal pain and aspects of lifestyle such as employment [12,13] and smoking [12]. However, another potentially important lifestyle factor, nutrition, has only been sparingly investigated. In the only studies to our knowledge concerning adolescent spinal pain and nutrition, Ghandour et al. [14] reported that back pain was related to high caffeine intake in adolescent girls, and Molcho et al. [15] documented that adolescents who reported going to bed hungry because there was insufficient food in the house were more likely to have back pain, even after adjustment for parental social class. Despite the dearth of information, it is likely that nutrition is an important factor relating to musculoskeletal health for adolescents, as healthy growth and development is nutrition dependent [16,17].

Furthermore, a possible link in adolescents is suggested by some adult evidence. Certain dietary patterns have been shown to reduce general pain sensitivity in adults, specifically greater intakes of sucrose [15-19] and omega 3 fatty acids [19], and lower intakes of saturated fats [19]. High post-prandial blood sucrose [20] and lipid concentrations [21] have also been linked to higher levels of pro-inflammatory factors. Conversely higher intakes of omega 3 fatty acids [22] are linked to lower levels of such factors, and it has been suggested that fruit and vegetables may reduce inflammatory processes through an anti-oxidant effect [23]. Although such influences on inflammation are likely to be short-term in themselves, effects are likely to be continuous, as food intake is repeated [23].

There is evidence that adolescent diets deviate further from recommended dietary guidelines than adult diets [24] and so nutritional effects on spinal pain may possibly be more common in adolescents. Contemporary adolescent diet in the western world is below recommended standards, with high intakes of sugar [25,26] and fat [25], low intakes of fibre [25,27] and a steady reduction in fruit and vegetable consumption throughout adolescence [28].

Altogether these findings suggest that there may be links between spinal pain and nutrition in adolescents. Given the lack of prior research in this area, our study aims to be exploratory in nature, and to investigate the links between a range of nutritional and dietary factors and adolescent spinal pain. Based on the limited prior evidence, the first hypothesis was that low or high consumption of specific nutrients or food groups may be associated with spinal pain. As individual nutrients or foods may have a very small impact, our second hypothesis was that adolescents with spinal pain will have a poorer quality diet and dietary pattern.

## Methods

### Design

This was a cross-sectional exploratory study conducted in Western Australia.

### Participants

Data from 1424 adolescents (696 girls, 728 boys) of mean (SD) height 1.64 (0.08) m, weight 57.6 (13.1) kg and age 14.06 (0.20) yrs were collected as part of their participation in the Western Australian Pregnancy Cohort 'Raine' Study http://www.rainestudy.org.au. There were 2337 adolescents eligible for the 14 year follow-up, of which 1704 (72.9%) consented to some aspect of the follow-up and 1424 (60.9%) completed the data collection requirements for the analysis reported in this paper. The initial cohort over-represented socially disadvantaged groups and there has been greater attrition in these groups so the remaining participants more closely match the Australian adolescent population [29,30]. Informed consent to participate in the 14 year follow-up was obtained from the primary carer. The study was approved by the Human Research Ethics Committees of Curtin University of Technology and Princess Margaret Hospital.

### Procedure

#### Nutritional data

At around the time of the adolescent's 14^th ^birthday a food frequency questionnaire (FFQ) was sent to the participants' homes by post for completion by the adolescent's carer in association with the study adolescent in order to assess the usual dietary intake of each adolescent. The FFQ is a semi-quantitative assessment developed by the Commonwealth Scientific and Industrial Research Organisation (CSIRO) in Australia [31]. The FFQ asks about the usual frequency of consumption of 212 food and beverage items, excluding alcohol, and how their usual serve (portion) compared to a standard serve size given in household measurements (spoons, cups, slices, etc). The questionnaires were returned in a pre-paid envelope and checked by a research nurse and missing or unclear responses were clarified when the adolescent attended their physical assessment. The CSIRO entered and verified the FFQs and provided estimates of daily intakes of foods and nutrients using Australian food composition data. This FFQ has been validated [32,33] and shown to correctly rank a reasonable proportion of most nutrient intakes when compared to a 3-day food record in this adolescent cohort [34].

#### Spinal pain data

Each adolescent was invited to a research centre to complete a questionnaire on a variety of developmental and health issues, some of which are reported in this paper. Spinal pain questions were: Has your back been painful in the last month? ('yes' or 'no'), and Has your neck/shoulder been painful in the last month? ('yes' or 'no'), The spinal pain questions were based on prior questions with demonstrated test-retest reliability and validity in comparison to clinical interview[8,35,36].

### Potential confounding variables

Because poor diet may relate to lower socioeconomic status in adolescence [37,38], which may in turn be related to subsequent spinal pain [39], this study also considered primary carer highest educational levels. As smoking [40] and activity [41] may also relate to both diet and spinal pain, they were also evaluated in analyses. Body fatness has been associated with back pain in adolescents [1,42] and was assessed by waist girth. Males and females were considered separately as both diet [43,44] and spinal pain [1,45] may differ between genders.

### Data management

As this was an exploratory study and prior literature had suggested possible links between spinal pain and specific nutrients, particular food groups and diet quality, the nutrition data were characterized in four ways.

#### a. Specific nutrients

The FFQ raw data were used by CSIRO to derive estimates of the intakes of specific nutrients. The nutrient content of consumed food was estimated from Australian nutrient databases [46,47], British food tables [48], and US Dept of Agriculture food tables [49]. An estimate of total energy intake was also derived.

#### b. Broad Food Groups

The FFQ was also processed by CSIRO to provide estimates of the intake of 212 foods which we combined into 16 food groups. Estimated consumption of these food groups was reported as grams per day.

#### c. Diet Quality scores

Five major food groups (cereal, vegetables, fruit, dairy products, meat and meat alternatives), six macronutrients (total fat, saturated fat, protein, fibre, iron, calcium) and 'extras' (which were sweet and salty snack foods including confectionery, biscuits and crisps) were further analysed by creating diet quality categories: 'least optimal', 'semi-optimal' and 'optimal' based on recommended serves of these food groups [50].

#### d. Dietary Pattern

To further characterize the type of diet a factor analysis of the food groups was also carried out [51]. Two main dietary types were found; a 'healthy' dietary pattern characterised by high loadings for fresh fruit, vegetables, wholegrains, legumes and fish (grilled, steamed or tinned only); and a 'western' dietary pattern loaded highly for take-away foods, red meat, processed meat, refined grains, hot chips, soft drinks, confectionery, cakes and biscuits, and high fat dairy products. Each adolescent received a z-score for both dietary patterns.

### Data analysis

Gender differences were analysed using independent t tests for each of the continuous variables, and Chi squared tests for the categorical variables. The continuous nutritional variables of specific nutrients and broad food groups were banded into the bottom 25%, inter-quartile range and top 25%. The diet quality variables were already in categories ('least optimal', 'semi-optimal' and 'optimal'). The continuous z scores for the two dietary patterns ('western' and 'healthy') were banded into four quartiles. The use of category bands allowed the analysis to detect non-linear relationships, for example where both high and low intakes of a food group was associated with spinal pain. Simple linear analysis would miss such an association.

Univariate logistic regression models predicting back or neck/shoulder pain during the past month for each gender from each nutrition characteristic were calculated, with statistical significance set at p < 0.05.

Forward stepwise likelihood ratio multivariate logistic regression models were used to evaluate the combined associations of nutritional factors, with the probability for entry and removal of covariates based on the likelihood ratio score statistic being p = 0.05 and 0.10 respectively. These criteria for inclusion/exclusion were set to balance false positive and false negative findings. The multivariate analyses of associations between spinal pain and nutrition were performed on specific nutrients and broad food categories. No multivariate analysis level was performed for diet quality categories or dietary patterns, as none were significant on univariate testing. Separate models were used for each of these levels, as well as for both genders. For each level, waist girth, primary carer education level, and adolescent smoking in past 12 months, were included in an initial step, with the banded nutritional variables from that analysis level that were significant in univariate testing included in a second step. Adolescent TV viewing and level of exercise outside school hours were unrelated to spinal pain, and thus were not included as potential confounders in multivariate analysis. All statistical analysis was performed using SPSS version 13.

### Ethics

This study was conducted according to the guidelines laid down in the Declaration of Helsinki and all procedures involving human subjects were approved by the Human Research Ethics Committees of Curtin University of Technology and Princess Margaret Hospital, Perth, Australia. Written, informed consent was obtained from all subjects.

## Results

### Spinal pain

Back or neck pain were experienced by a substantial portion of adolescents. 27.8% of adolescents reported back pain in the past month. Females (30%) had a trend (χ2 = 3.26, p = 0.071) for a slightly higher back pain prevalence than males (25.7%). Similarly, 28.7% of adolescents reported neck pain in the past month. Females had a greater (χ2 = 17.72, p < 0.001.) prevalence (33.9%) than males (23.8%). The gender differences in spinal pain were independent of levels of dietary factors. For example, the female gender was significantly associated with neck pain, with similar odds ratios before (OR 1.7, 95%CI: 1.4 to 2.2) and after (OR 1.6, 95%CI 1.3 to 2.1) adjustment for western diet type.

### Nutrition

Nutritional intakes were significantly different for males and females in the majority of specific nutrients and broad food groups (tables 1 and 2). Males tended to have a greater intake of nutrients and broad food groups, although females had a greater intake of vegetable products, soups and confectionary bars (table 2). The quality of nutrition, as measured by diet quality scores, also tended to differ across genders, with fewer females having an 'optimal' or 'semi-optimal' intake of cereals, dairy products, and calcium, and less males having an 'optimal' intake of fibre (table 3). Although significantly fewer females than males had optimal levels of iron and meat, this was a small difference (table 3). Fewer than half of adolescents had an 'optimal' intake of cereal, vegetables, dairy products, saturated fats, fats in general, 'extras' and fibre (table 3). This poor quality diet was more apparent in males, as shown by males having a significantly higher factor score for the 'western' dietary pattern (table 4).

**Table 1:** Specific nutrient intakes in males and females

	Males		Females		Gender difference	
	Mean	SD	Mean	SD	t, df	p
omega 3 - g/day	1.39	0.69	1.19	0.59	5.9, 1420	<0.001
omega 6 - g/day	12.43	6.51	10.80	5.71	5.0, 1420	<0.001
long chain n3 acids	0.27	0.13	0.23	0.13	5.2, 1420	<0.001
omega 3 fatty acid:2n6 (g)	12.13	6.47	10.54	5.68	4.9, 1420	<0.001
omega 3 fatty acid:2n6 (g)	0.02	0.01	0.02	0.01	6.4, 1420	<0.001
omega 3 fatty acid:3n3 (g)	1.12	0.63	0.93	0.51	5.9, 1420	<0.001
omega 3 fatty acid:3n6 (g)	0.04	0.02	0.03	0.02	6.3, 1420	<0.001
Arachidonic (g)	0.19	0.08	0.16	0.08	6.9, 1420	<0.001
omega 3 fatty acid:4n6 (g)	0.02	0.01	0.02	0.01	7.1, 1420	<0.001
eicosapentanoic acid (EPA) (g)	0.08	0.04	0.07	0.04	5.6, 1420	<0.001
omega 3 fatty acid:5n6 (g)	0.00	0.00	0.00	0.00	0.7, 1420	0.462
docosapentanoic acid (DPA) (g)	0.11	0.05	0.09	0.05	6.3, 1420	<0.001
docosahexanoic acid (DHA) (g)	0.08	0.06	0.07	0.06	2.2, 1420	0.030
water (g)	2,477	849	2,255	810	5.0, 1420	<0.001
total sugar (g)	169.17	64.22	145.99	64.05	6.8, 1420	<0.001
complex CHO (g)	128.54	44.70	105.32	36.47	10.7, 1420	<0.001
fibre (g)	25.01	9.78	23.09	8.81	3.9, 1420	<0.001
nitrogen (g)	16.97	5.04	14.09	4.74	11.1, 1420	<0.001
Kcalories	2,525	764	2,115	728	10.3, 1420	<0.001
Kjoules	10,519	3,186	8,813	3,034	10.3, 1420	<0.001
protein (g)	104.96	31.40	86.99	29.38	11.1, 1420	<0.001
total fat (g)	99.82	35.32	83.48	32.79	9.0, 1420	<0.001
total carbohydrate (g)	300.13	96.02	253.17	91.92	9.4, 1420	<0.001
sodium (mg)	3,607	1,147	3,041	1,086	9.5, 1420	<0.001
potassium (mg)	4,000	1,345	3,497	1,262	7.3, 1420	<0.001
calcium (mg)	1,349	597	1,042	502	10.5, 1420	<0.001
Magnesium (mg)	341.62	115.48	290.38	103.50	8.8, 1420	<0.001
phosphorous (mg)	1,823	626	1,479	553	10.9, 1420	<0.001
iron (mg)	15.07	5.05	12.62	4.29	9.8, 1420	<0.001
copper (mg)	2.05	0.66	1.81	0.63	6.9, 1420	<0.001
zinc (mg)	13.92	4.39	11.66	4.04	10.1, 1420	<0.001
retinol (ug)	582.79	521.65	450.43	501.37	4.9, 1420	<0.001
carotene (ug)	4,295	2,113	4,278	2,279	0.1, 1420	0.883
vitamin D (ug)	2.38	1.53	2.01	1.33	4.9, 1420	<0.001
thiamin (mg)	1.97	0.76	1.56	0.56	11.3, 1420	<0.001
riboflavin (mg)	2.76	1.10	2.16	0.89	11.3, 1420	<0.001
nicotinic acid (mg)	19.78	6.34	16.76	5.54	9.6, 1420	<0.001
pot.nicotinic acid (mg)	20.53	6.27	16.97	5.87	11.0, 1420	<0.001
vitamin C (mg)	186.00	108.46	190.91	119.31	-0.8, 1420	0.416
vitamin E (mg)	9.37	4.85	8.00	3.89	5.8, 1420	<0.001
vitamin b6 (mg)	1.87	0.70	1.67	0.62	5.6, 1420	<0.001
vitamin b12 (ug)	5.03	2.18	4.05	2.15	8.6, 1420	<0.001
free folate (ug)	151.90	68.26	142.81	67.14	2.5, 1420	0.011
total folate (ug)	258.49	101.71	238.50	97.63	3.8, 1420	<0.001
pantothenate (mg)	5.77	2.04	4.93	1.91	8.1, 1420	<0.001
biotin (ug)	25.27	10.15	20.44	8.79	9.6, 1420	<0.001
alcohol (g)	0.00	0.07	0.01	0.18	-0.6, 1420	0.557
cholesterol (mg)	334.35	143.14	272.22	126.77	8.6, 1420	<0.001
refined sugar (g)	80.17	41.75	69.18	41.82	5.0, 1420	<0.001
saturated fat (g)	44.40	17.53	36.24	16.04	9.2, 1420	<0.001
monounsaturated fat (g)	34.51	12.37	28.82	11.41	9.0, 1420	<0.001
polyunsaturated fat (g)	14.72	7.33	12.85	6.28	5.2, 1420	<0.001
total nicotinic acid (mg)	40.31	12.09	33.72	11.06	10.7, 1420	<0.001
total vitamin A (ug)	1,298.76	661.95	1,163.54	669.46	3.8, 1420	<0.001
natural sugars (g)	89.00	42.93	76.81	37.05	5.7, 1420	<0.001

**Table 2:** Broad food intakes in males and females (grams/day)

	Males		Females		Gender difference	
	Mean	SD	Mean	SD	t, df	p
Non-alcoholic beverages*	1,185.2	622.1	1,164	585	0.6, 1420	0.521
Cereals and cereal products	260.75	109.5	219.81	91.73	7.6, 1420	<0.001
Cereal-based products	90.34	58.05	68.24	44.71	8.0, 1420	<0.001
Fats and oils	23.59	13.05	19.42	9.87	6.8, 1420	<0.001
Fish and seafood products	80.95	100.0	71.21	82.44	2.0, 1420	0.046
Fruit products	255.18	220.5	255.51	187.07	0.0, 1420	0.976
Egg products	17.65	16.60	15.33	15.48	2.7, 1420	0.006
Meat, poultry and game products	185.90	78.60	158.73	76.84	6.6, 1420	<0.001
Milk products	637.39	372.3	435.16	288.49	11.4, 1420	<0.001
Soups	18.99	21.80	23.88	28.68	-3.6, 1420	<0.001
Seed and nut products	2.17	5.66	2.18	6.08	0.0, 1420	0.966
Savoury sauces and condiments	12.13	11.80	10.85	12.13	2.0, 1420	0.044
Vegetable products	109.35	64.86	118.83	64.67	-2.8, 1420	0.006
Legume and pulse products	9.01	14.81	7.67	12.96	1.8, 1420	0.070
Snack foods	17.84	14.17	16.43	13.97	1.9, 1420	0.059
Sugar products	12.26	16.30	9.26	10.80	4.1, 1420	<0.001
Confectionery and health bars	45.10	35.42	46.25	41.61	-0.6, 1420	0.574
Special dietary foods	2.89	20.85	1.64	21.34	1.1, 1420	0.265
Miscellaneous	15.38	18.95	14.25	26.15	0.9, 1420	0.352

**Table 3:** Diet quality scores in males and females (percentage meeting least optimal, semi-optimal and optimal intakes)

	Males			Females			Gender difference	
Food score	Least optimal	Semi- optimal	Optimal	Least optimal	Semi- optimal	optimal	Chi Sq	p
Cereal Score	12.5	58.2	29.3	25.7	61.5	12.8%	79.8	<0.001
Vegetable Score	57.3	37.2	5.4	52.3	40.1	7.6%	5.0	0.081
Fruit Score	23.1	23.1	53.8	19.8	26.1	54.0%	3.1	0.208
Dairy Score	24.3	44.3	31.4	47.1	38.9	13.9%	102.6	<0.001
Meat Score	0.1	2.4	97.4	0.7	4.9	94.4%	9.1	0.011
Extra Score	32.6	32.3	35.1	24.6	27.0	48.4%	26.9	<0.001
Fat Score	0.5	83.0	16.4	1.0	82.6	16.4%	1.0	0.606
Saturated Fat score	56.1	38.9	5.0	50.4	44.3	5.3%	4.7	0.094
Protein Score	0.0	2.0	98.0	0.0	1.29	98.7%	1.2	0.272
Fibre Score	9.2	58.4	32.3	4.2	47.1	48.7%	46.0	<0.001
Calcium Score	4.6	30.3	65.1	11.4	46.8	41.8%	82.1	<0.001
Iron Score	0.0	4.6	95.4	0.0	12.64	87.4%	29.6	<0.001

**Table 4:** Dietary pattern in males and females

	Males		Females		Gender difference	
	Mean	SD	Mean	SD	t, df	p
Healthy type	-0.047	0.91	0.128	0.85	-1.3, 1416	0.202
Western type	0.164	0.86	-0.179	0.88	7.4, 1416	<0.001

### Nutrition and Spinal Pain

In univariate analysis only a few dietary variables were associated with spinal pain. In females, a reduced risk of back pain was associated with high intakes of some nutritional elements (meat, sodium, copper, carotene and vitamin B6) and with low intakes of others (vitamin E, polyunsaturated fat and omega 6 fatty acids). Similarly for females there was a reduced risk of neck/shoulder pain associated with high (fruits and meat) and low intakes (vitamin B12 and 5n3 DPAs). In males, a reduced risk of back pain was associated with high intakes (fruits) and low intakes of some nutritional elements (iron and nicotinic acid). An increased risk of back pain was associated with highest intakes of fish and seafood in males. A reduced risk of neck/shoulder pain was associated with highest intakes of egg products and sugar products, whilst an increased risk was associated with lowest intakes of cereals, retinol, vitamin D, vitamin B12, and omega 6 fatty acids. None of the other nutrition variables showed significant associations with back or neck/shoulder pain.

After multivariate analysis at the different levels of nutrition characterization there were only a few associations found with back and neck pain (table 5) and these varied across pain location and gender.

**Table 5:** Statistically significant multivariate relationships between nutrition variables and back and neck pain in males and females.

Gender		Nutrition variables	OR	95% CIs	
		**Back pain**			

Female	Egg Products	<3.67 g	0.46	0.29	0.75
		3.67-22.15 g	reference		
		>22.15 g	0.96	0.63	1.47
		**Neck Pain**			

Male	Cereal Score	<186.3 g	1.62	1.03	2.54
		186.3 - 311.2 g	reference		
		>311.2 g	1.61	1.03	2.54
Female	Vitamin B12	<2.74 μg	1.72	1.14	2.62
		2.74-4.96 μg	reference		
		>4.96 μg	1.24	0.81	1.910
	Meat Score	<106.9 g	1.14	0.754	1.73
		106.9 - 198.2 g	reference		
		>198.2 g	0.62	0.40	0.97

The nutrition variables are categorised into lowest 25%, interquartile range, and highest 25%

Footnote: Forward stepwise likelihood ratio multivariate logistic regression models adjusting for waist girth, primary carer education level and adolescent smoking in past 12 months

## Discussion

This study confirms that spinal pain is clearly a significant problem for adolescents, concurring with previous findings [1,2], and underlining the importance of research into possible risk factors.

Whilst the majority of our sample had more than adequate intakes of meat, protein and iron, there were significant numbers of adolescents failing to have sufficient intakes of cereals, fruit, dairy products, vegetables, fibre or calcium, and almost all had excessive consumption of saturated fats and snack foods. These findings are similar to those recently observed in other Australian adolescents [52].

In terms of associations between spinal pain and specific nutrients and food groups - vitamin B12, egg, cereal and meat consumption were related to spinal pain. For females low egg consumption and high meat consumption were related to a reduced risk of back and neck pain respectively. A low intake of vitamin B12 was related to an increased risk of neck pain in females. For males both low and high consumption of cereals were related to increased risk of neck pain. This study cannot identify specific mechanisms for the effect of nutrition on adolescent spinal pain, but previous adult and animal literature suggests several possibilities including effects on pain sensitivity [19] and inflammation [23] which could partially explain our findings.

A noticeable feature of the specific nutrient and food group relationships with spinal pain was the lack of any consistency across pain locations, or genders. This may reflect real differences; for example, some types of neck and back pain may have differing mechanisms [53], and males and females may have different pain mechanisms [54]. In addition, the lack of consistency probably reflects the fact that any individual nutrient, in contrast to a dietary pattern, may usually only contribute minimally to any effects on spinal pain. These results thus provide weak support for our first study hypothesis that specific nutrients or food groups are related to adolescent spinal pain.

In terms of associations between spinal pain and diet quality and dietary pattern, there were no significant univariate results. These results therefore do not support our second hypothesis that diet quality and dietary pattern are related to adolescent back pain.

The lack of further significant findings was not due to an under-powered study as the majority of the significant relationships detected were weak. Furthermore, the weak individual nutrient relationships were not due to lack of consideration of curvilinear relationships as we analysed for both high and low consumption. They were also not due to a lack of adequate characterization of diet (considered at 4 levels - specific nutrients, good groups, diet quality and dietary pattern), lack of consideration of different gender relationships, or a lack of adjustment for confounding by physical activity, smoking, body fatness and socioeconomic status, as all these were considered in the study design. The weak evidence for relationships between diet and spinal pain may have been due to our characterization of spinal pain. We did not specify the area of back pain with a diagram for participants. However we had similar findings when we repeated the analyses using self-reported back and neck pain ever and chronic back and neck pain (lasting more than 3 months) and additionally when using a parental report of health professional diagnosed back or neck pain (data not shown). Our relatively stringent multivariate inclusion/exclusion criteria may also have meant our analysis missed some weaker nutrition relationships. One further possible cause of the weak relationships may have been a failure to distinguish between sub-groups of spinal pain with different aetiologies.

Whilst our findings suggest that diet in adolescence may be related to spinal pain, the cross-sectional nature of the findings does not allow causality to be assumed. Further longitudinal studies should be undertaken to examine causality. If diet is shown to contribute to spinal pain, then the burden of adolescent spinal pain may be lessened by greater and more successful attempts to improve nutrition in adolescents. Furthermore, because adolescent spinal pain is a risk factor for spinal pain, improvements in adolescent diet may have beneficial effects into adulthood, reducing the overall burden of spinal pain on society.

## Conclusions

This study found that many Western Australian adolescents had non-optimal diets. The findings of this exploratory study suggest that certain aspects of diet (Vitamin B12, eggs, cereals and meat) may have an association with spinal pain in adolescence. These results provide important initial evidence that diet and adolescent spinal pain may be associated, but further work is needed to explore potential relationships and mechanisms.

## Competing interests

The authors declare that they have no conflicts of interest.

## Authors' contributions

Authors have contributed in the following ways: MP analysed the data, performed the literature review, wrote the first draft of the manuscript and updated the manuscript after co-author feedback. LS initiated the spinal pain aspect of the study, assisted with data analysis and contributed to reviewing and subsequent improvements of the manuscript. WO initiated the nutrition aspect of the study and nutrition data collection, assisted with data analysis, contributed to reviewing the drafts and improving the manuscript. PO helped to initiate the spinal pain aspect of the study and contributed to reviewing and improvements of the manuscript. AS was significantly involved in the statistical analysis and contributed to the reviewing and improvements of the manuscript drafts. All authors read and approved final manuscript.

## Pre-publication history

The pre-publication history for this paper can be accessed here:

http://www.biomedcentral.com/1471-2474/11/138/prepub
